# New Tools and Connections for Exponential-Time Approximation 

**DOI:** 10.1007/s00453-018-0512-8

**Published:** 2018-09-05

**Authors:** Nikhil Bansal, Parinya Chalermsook, Bundit Laekhanukit, Danupon Nanongkai, Jesper Nederlof

**Affiliations:** 1 Eindhoven University of Technology, Eindhoven, The Netherlands; 2 Aalto University, Helsinki, Finland; 3 Shanghai University of Finance and Economics, Shanghai, China; 4 KTH, Royal Institute of Technology, Stockholm, Sweden

**Keywords:** Approximation algorithms, PCP’s, Exponential time algorithms

## Abstract

In this paper, we develop new tools and connections for *exponential time approximation*. In this setting, we are given a problem instance and an integer $r>1$, and the goal is to design an approximation algorithm with the fastest possible running time. We give randomized algorithms that establish an approximation ratio of

1. *r* for maximum independent set in $O^*(\exp ({\tilde{O}}(n/r \log ^2 r+r\log ^2r)))$ time,
2. *r* for chromatic number in $O^*(\exp (\tilde{O}(n/r \log r+r\log ^2r)))$ time,
3. $(2-1/r)$ for minimum vertex cover in $O^*(\exp (n/r^{\varOmega (r)}))$ time, and
4. $(k-1/r)$ for minimum *k*-hypergraph vertex cover in $O^*(\exp (n/ (kr)^{\varOmega (kr)}))$ time.

*r* for maximum independent set in $O^*(\exp ({\tilde{O}}(n/r \log ^2 r+r\log ^2r)))$ time,

*r* for chromatic number in $O^*(\exp (\tilde{O}(n/r \log r+r\log ^2r)))$ time,

$(2-1/r)$ for minimum vertex cover in $O^*(\exp (n/r^{\varOmega (r)}))$ time, and

$(k-1/r)$ for minimum *k*-hypergraph vertex cover in $O^*(\exp (n/ (kr)^{\varOmega (kr)}))$ time.

(Throughout, ${\tilde{O}}$ and $O^*$ omit $\hbox {polyloglog} (r)$ and factors polynomial in the input size, respectively.) The best known time bounds for all problems were $O^*(2^{n/r})$ (Bourgeois et al. in Discret Appl Math 159(17):1954–1970, 2011; Cygan et al. in Exponential-time approximation of hard problems, 2008). For maximum independent set and chromatic number, these bounds were complemented by $\exp (n^{1-o(1)}/r^{1+o(1)})$ lower bounds (under the Exponential Time Hypothesis (ETH)) (Chalermsook et al. in Foundations of computer science, FOCS, pp. 370–379, 2013; Laekhanukit in Inapproximability of combinatorial problems in subexponential-time. Ph.D. thesis, 2014). Our results show that the naturally-looking $O^*(2^{n/r})$ bounds are not tight for all these problems. The key to these results is a *sparsification* procedure that reduces a problem to a bounded-degree variant, allowing the use of approximation algorithms for bounded-degree graphs. To obtain the first two results, we introduce a new *randomized branching rule*. Finally, we show a connection between PCP parameters and exponential-time approximation algorithms. This connection together with our independent set algorithm refute the possibility to overly reduce the size of Chan’s PCP (Chan in J. ACM 63(3):27:1–27:32, 2016). It also implies that a (significant) improvement over our result will refute the gap-ETH conjecture (Dinur in Electron Colloq Comput Complex (ECCC) 23:128, 2016; Manurangsi and Raghavendra in A birthday repetition theorem and complexity of approximating dense CSPs, 2016).

## Introduction

The Independent Set, Vertex Cover, and Coloring problems are central problems in combinatorial optimization and have been extensively studied. Most of the classical results concern either approximation algorithms that run in polynomial time or exact algorithms that run in (sub)exponential-time. While these algorithms are useful in most scenarios, they lack flexibility: Sometimes, we wish for a better approximation ratio with worse running time (e.g., computationally powerful devices), or faster algorithms with less accuracy. In particular, the trade-offs between the running time and approximation ratios are needed in these settings.

Algorithmic results on the trade-offs between approximation ratio have been studied already in the literature in several settings, most notably in the context of *Polynomial-time Approximation Schemes (PTAS)*. For instance, in planar graphs, Baker’s celebrated approximation scheme for several NP-hard problems [2] gives an $(1+\varepsilon )$-approximation for, e.g., Independent Set in time $O^*(\exp (O(1/\varepsilon )))$ time. In graphs of small treewidth, Czumaj et al. [16] give an $O^*(\exp (tw/r))$ time algorithm that given a graph along with a tree decomposition of it of width at most *tw*, find an *r*-approximation for Independent Set. For general graphs, approximation results for several problems have been studied in several works (see, e.g., [6–8, 13–15]). A basic building block that lies behind many of these results is to partition the input instance in smaller parts in which the optimal (sub)solution can be computed quickly (or at least faster than fully exponential-time). For example, to obtain an *r*-approximation for Independent Set one may arbitrarily partition the vertex set in *r* blocks and restrict attention to independent sets that are subsets of these blocks to get a $O^*(\exp (n/r))$ time *r*-approximation algorithm.

While at first sight one might think that such a naïve algorithm should be easily improvable via more advanced techniques, it was shown in [6, 11] that almost linear-size PCPs with sub-constant error imply that *r*-approximating Independent Set [11] and Coloring [31] requires at least $\exp (n^{1-o(1)}/r^{1+o(1)})$ time assuming the popular Exponential Time Hypothesis (ETH). In the setting of the more sophisticated Baker-style approximation schemes for planar graphs, Marx [34] showed that no $(1+\varepsilon )$-approximating algorithm for planar Independent Set can run in time $O^*(\exp ((1/\varepsilon )^{1-\delta }))$ assuming ETH, which implies that the algorithm of Czumaj cannot be improved to run in time $O^*(\exp (tw/r^{1+\varepsilon }))$.

These lower bounds, despite being interesting, do not say anything about the lower order terms and by no means answer the question whether the known approximation trade-offs can be improved significantly, and in fact in many settings we are far from understanding the full power of exponential time approximation. For example, until recently [10], we cannot exclude (under any plausible complexity assumption) algorithms that 2-approximate *k*-Independent Set^1^ in time $n^{o(k)}$ (see also [30]), nor do we know algorithms that run asymptotically faster than the fastest exact algorithm that runs in time $n^{0.792k}$ time [36].

In this paper, we aim to advance this understanding and study the question of designing fast (exponential-time) algorithms that guarantee the designated approximation ratios of *r* for Independent Set, Coloring and Vertex Cover in general (hyper)graphs. Ultimately, we wish to design approximation algorithms that are as fast as possible.

### Our Results

For Independent Set, our result is the following. Here we use $\tilde{O}$ to omit log log factors in *r*.

#### Theorem 1

There is a randomized algorithm that given an *n*-vertex graph *G* and integer *r* outputs an independent set that, with constant positive probability, has size at least $\alpha (G)/r$, where $\alpha (G)$ denotes the maximum independent set size of *G*. The algorithm runs in expected time $O^*(\exp (\tilde{O}(n/(r\log ^2 r)+r \log ^2 r)))$.

To prove this result, we introduce a new *randomized branching rule* that we will now introduce and put in context towards previous results. This follows a *sparsification technique* that reduces the maximum degree to a given number. This technique was already studied before in the setting of exponential time approximation algorithms for Independent Set by Cygan et al. (see [13, paragraph ‘Search Tree Techniques’]) and Bourgeois et al. (see [8, Section 2.1]), but the authors did not obtain running times sub-exponential in *n* / *r*. Specifically, the sparsification technique is to branch (e.g., select a vertex and try to both include *v* in an independent set or discard and recurse for both possibilities) on vertices of sufficiently high degree. The key property is that if we decide to include a vertex in the independent set, we may discard all neighbors of *v*. If we generate instances by keeping branching on vertices of degree at least *d* until the maximum degree is smaller than *d*, then at most $$\left( {\begin{array}{c}n\\ n/d\end{array}}\right) \lessapprox \exp (n\log (d)/d)$$ instances are created. In each such instance, the maximum independent set can be easily *d*-approximated by a greedy argument. Cygan et al. [13] note that this gives worse than $O^*(2^{n/r})$ running times.

Our algorithm works along this line but incorporates two (simple) ideas. Our first observation is that instead of solving each leaf instance by greedy *d*-approximation algorithm, one can use a recent ${\tilde{O}}(\frac{d}{\log ^2 d})$ approximation algorithm by Bansal et al. [3] for Independent Set on bounded degree graphs. If we choose $d \approx r \log ^2 r$, this immediately gives an improvement, an *r*-approximation in time essentially $\mathsf{exp}(\frac{n}{r \log r})$. To improve this further, we present an additional (more innovative) idea introducing randomization. This idea relies on the fact that in the sparsification step we have (unexploited) slack as we aim for an approximation.^2^ Specifically, whenever we branch, we only consider the ‘include’ branch with probability 1 / *r*. This will lower the expected number of produced leaf instances in the sparsification step to $2^{n/d} \approx \mathsf{exp}(\frac{n}{r \log ^2 r})$ and preserves the approximation factor with good probability.

Via fairly standard methods (see, e.g., [5]) we show this also gives a faster algorithm for coloring in the following sense:

#### Theorem 2

There is a randomized algorithm that, given an *n*-vertex graph *G* and an integer $r>0$, outputs with constant positive probability a proper coloring of *G* using at most $r\cdot \chi (G)$ colors. The algorithm runs in time $O^*(\exp (\tilde{O}(n/(r\log r)+r \log ^2 r)))$.

As a final indication that sparsification is a very powerful tool to obtain fast exponential time approximation algorithms, we show that a combination of a result of Halperin [22] and the sparsification Lemma [25] gives the following result for the Vertex Cover problem in hypergraphs with edges of size at most *k* (a.k.a. the Set Cover problem with frequency at most *k*).

#### Theorem 3

For every *k*, there is an $r_0:=r(k)$ such that for every $r \ge r_0$ there is an $O^*(\exp (\frac{n}{(kr)^{\varOmega (kr)}}))$ time randomized $(k-\tfrac{1}{r})$-approximation algorithm for the Vertex Cover problem in hypergraphs with edges of size at most *k*.

Note that for $k=2$ (e.g., vertex cover in graphs), this gives an $O^*(\exp (\frac{n}{r^{\varOmega (r)}}))$ running time, which gives an exponential improvement (in the denominator of the exponent) upon the $(2-1/r)$ approximation by Bonnet et al. [8] that runs in time $O^*(2^{n/r})$. It was recently brought to our attention that Williams and Yu [38] independently have unpublished results for (hypergraph) vertex cover and independent set using sparsification techniques similar to ours.

*Connections to PCP parameters* The question of approximating the maximum independent set problem in sub-exponential time has close connections to the trade-off between three important parameters of PCPs: *size*, *gap* and *free-bit*. We discuss the implications of our algorithmic results in terms of these PCP parameters.

Roughly speaking, the gap parameter is the ratio of *completeness* to *soundness*, while the *freeness* parameter is the number of “locally” distinct proofs that would cause the verifier to accept 3; the *free-bit* is simply a logarithm of freeness. For convenience, we will continue our discussions in terms of freeness, instead of freebit.

- *Freebit versus Gap* The dependency between freeness and gap has played an important role in hardness of approximation. Most notably, the existence of PCPs with freeness $g^{o(1)}$ where *g* is a gap parameter is “equivalent” to $n^{1-o(1)}$ hardness of approximating maximum independent set [4, 23]; this result is a building block for proving other hardness of approximation for many other combinatorial problems, e.g., coloring [20], disjoint paths [1], induced matching [11], cycle packing [21], and pricing [11]. Arguably, the trade-off of these PCP parameters captures the approximability of many natural combinatorial problems.
  Better parameter trade-off implies stronger hardness results. The existence of a PCP with arbitrarily large gap, freeness 1 (lowest possible), and completeness close to 1 / 2, is in fact equivalent to $(2-\epsilon )$ inapproximability for Vertex Cover [4]. The best known trade-off is due to Chan [12]: For any $g >0$, there is a polynomial-sized PCP with gap *g* (completeness close to one) and freeness $O(\log g)$, yielding the best known NP-hardness of approximating maximum independent set in sparse graphs, i.e., $\varOmega (d/ \log ^4 d)$ NP-hardness of approximating maximum independent set in degree-*d* graphs. 4
- *Size, Freebit, and Gap* When a polynomial-time approximation algorithm is the main concern, polynomial size PCPs are the only thing that matter. But when it comes to exponential time approximability, another important parameter, *size* of the PCPs, has come into play. The trade-off between size, freebit, and gap tightly captures the (sub-)exponential time approximability of many combinatorial problems. For instance, for any constant $g >0$, Moshkovitz and Raz [35] construct PCPs^5^ of size $n^{1+o(1)}$ and freeness $2^{O(\sqrt{\log g})}$ and gap *g*; this implies that *r*-approximating Independent Set requires time $2^{n^{1-o(1)}/r^{1+o(1)}}$ [11].

Our exponential-time approximation result for Independent Set implies the following trade-off results.

#### Corollary 1

Unless the ETH fails, a freebit PCP on an *n*-variable SAT formula, with gap parameter *g*, freeness parameter *F* and size parameter *S* must satisfy $F \cdot S = \varOmega (n \log ^2 g)$.

In particular, this implies that (i) the size of Chan’s PCP cannot be made smaller than $o(n \log g)$ unless ETH breaks, and (ii) in light of the equivalence between gap-amplifying freebit PCPs with freeness 1 and $(2-\epsilon )$ approximation for Vertex Cover, our result shows that such a PCP must have size at least $\varOmega (n \log ^2 g)$. We remark that no such trade-off results are known for polynomial-sized PCPs. To our knowledge, this is the first result of its kind.

*Further Related Results* The best known results for Independent Set in the polynomial-time regime are an $O(\frac{n (\log \log n)^2}{\log ^3 n})$-approximation [18], and the hardness of $n/\mathsf{exp}(O(\log ^{3/4+ o(1)} n))$ (which also holds for Coloring) [28]. For Vertex Cover, the best known hardness of approximation is $(\sqrt{2} - o(1))$ NP-hardness [26, 27] and $(2-\epsilon )$ hardness assuming the unique games conjecture [29]. All three problems (Independent Set, Coloring, and Vertex Cover) do not admit exact algorithms that run in time $2^{o(n)}$, unless ETH fails. Besides the aforementioned works [8, 13] sparsification techniques for exponential time approximation were studied by Bonnet and Paschos in [7], but mainly hardness results were obtained.

## Preliminaries

We first formally define the three problems that we consider in this paper. Independent Set: Given a graph $G=(V,E)$, we say that $J \subseteq V$ is an independent set if there is no edge with both endpoints in *J*. The goal of Independent Set is to output an independent set *J* of maximum cardinality. Denote by $\alpha (G)$, the cardinality of a maximum independent set. Vertex Cover: Given a graph $G=(V,E)$, we say that $J \subseteq V$ is a vertex cover of *G* if every edge is incident to at least one vertex in *J*. The goal of Vertex Cover is to output a vertex cover of minimum size. A generalization of vertex cover, called *k*-Hypergraph Vertex Cover (*k*-Vertex Cover),^6^ is defined as follows. Given a hypergraph $G=(V,{{\mathcal {E}}})$ where each hyperedge $h \in {{\mathcal {E}}}$ has cardinality at most *k*, the goal is to find a collection of vertices $J \subseteq V$ such that each hyperedge is incident to at least one vertex in *J*, while minimizing |*J*|. The *degree *$\varDelta (H)$ of hypergraph *H* is the maximum frequency of an element. Coloring: Given a graph $G=(V,E)$, a proper *k*-coloring of *G* is a function $f: V \rightarrow [k]$ such that $f(u) \ne f(v)$ for all $uv \in E$. The goal of Coloring is to compute a minimum integer $k>0$ such that *G* admits a (proper) *k*-coloring; this number is referred to as the *chromatic number*, denoted $\chi (G)$.

For a graph $G=(V,E)$, $N_G(v)$ denotes the set of neighbors of *v* and $d_G(v)$ denotes $|N_G(v)|$. If $X \subseteq V$ we let *G*[*X*] denote the graph $(X, E \cap (X \times X))$, i.e., the subgraph of *G* induced by *X*. We use $\exp (x)$ to denote $2^x$ in order to avoid superscripts. We use the $O^*(\cdot )$-notation to suppress factors polynomial in the input size. We use $\tilde{O}$ and ${\tilde{\varOmega }}$ to suppress factors polyloglog in *r* in respectively upper and lower bounds and write ${\tilde{\Theta }}$ for all functions that are in both $\tilde{O}$ and ${\tilde{\varOmega }}$.

## Faster Approximation via Randomized Branching and Sparsification

### Maximum Independent Set

In this section, we prove Theorem 1. Below is our key lemma.

#### Lemma 1

Suppose there is an approximation algorithm $\mathtt {dIS}(G,r)$ that runs in time *T*(*n*, *r*) and outputs an independent set of *G* of size $\alpha (G)/r$ if *G* has maximum degree *d*(*r*), (where $d(r) \ge 2r$). Then there is an algorithm $\mathtt {IS}(G,r)$ running in expected time $O^*(\exp (\tfrac{n}{d(r)}\log (4d(r)/r))T(n,r))$ that outputs an independent set of expected size $\alpha (G)/r$.

**Fig. 1:**
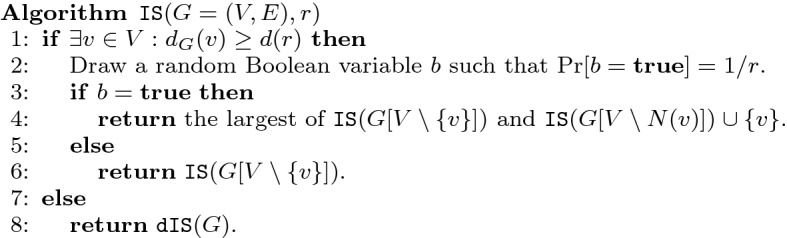
Approximation algorithm for the maximum independent set problem using an approximation algorithm *dIS* that works in bounded degree graphs

#### Proof

Consider the algorithm listed in Fig. 1.

For convenience, let us fix *r* and $d:=d(r)$. We start by analyzing the expected running time of this algorithm. Per recursive call the algorithm clearly uses $O^*(T(n,r))$ time. It remains to bound the expected number of recursive calls *R*(*n*) made by $\mathtt {IS}(G,r)$ when *G* has *n* vertices. We will bound $R(n) \le 2^{\lambda n}$ for $\lambda = \log (4d/r)/d$ by induction on *n*. Note that here $\lambda$ is chosen such that

1 $$\begin{aligned} \exp (-\lambda \cdot d) = r/(4d) \le \frac{r\log (4d/r)}{2d}, \end{aligned}$$

where we use $d/r \ge 2$ for the inequality. For the base case of the induction, note that if the condition at Line 1 does not hold, the algorithm does not use any recursive calls and the statement is trivial as $\lambda$ is clearly positive. For the inductive step, we see that

$$\begin{aligned} R(n)&\le R(n-1) + \Pr [b=\mathbf {true} ]\cdot R(n-d)&\\&= R(n-1) + R(n-d)/r&\\&= \exp (\lambda (n-1))+\exp (\lambda (n-d))/r&\\&= \exp (\lambda n)\left( \exp (-\lambda )+\exp (-\lambda d)/r\right)&\hbox { Using } \exp (-x) \le 1-x/2\hbox { for } x \in [0,1]\\&\le \exp (\lambda n)\left( 1-\lambda /2+\exp (-\lambda d)/r\right)&\hbox {Using }\exp (-\lambda \cdot d(r)) \le \lambda r/2\hbox { from }(1) \\&\le \exp (\lambda n). \end{aligned}$$

We continue by analyzing the output of the algorithm. It clearly returns a valid independent set as all neighbors of *v* are discarded when *v* is included in Line 4 and an independent set is returned at Line 8. It remains to show $\mathbb {E}[|\mathtt {IS}(G,r)|] \ge \alpha (G)/r$ which we do by induction on *n*. In the base case in which no recursive call is made, note that on Line 8 we indeed obtain an *r*-approximation as *G* has maximum degree *d*(*r*). For the inductive case, let *X* be a maximum independent set of *G* and let *v* be the vertex as picked on Line 1. We distinguish two cases based on whether $v \in X$. If $v \notin X$, then $\alpha (G)=\alpha (G[V\setminus v])$ and the inductive step follows as $\mathbb {E}[|\mathtt {IS}(G[V \setminus v],r)|] \ge \alpha (G)/r$ by the induction hypothesis. Otherwise, if $v \in X$, then $\mathbb {E}[|\mathtt {IS}(G,r)|]$ is at least

$$\begin{aligned}&\Pr [b=\mathbf {false} ]\cdot \mathbb {E}[|\mathtt {IS}(G[V {\setminus } \{v\}],r)|]+\Pr [b=\mathbf {true} ]\cdot \mathbb {E}[|\mathtt {IS}(G[N {\setminus } N(v)],r)|+1]\\&\quad \ge \left( 1-\tfrac{1}{r}\right) \frac{\alpha (G)-1}{r} + \tfrac{1}{r}\left( \frac{\alpha (G)-1}{r}+1\right) \\&\quad = \frac{\alpha (G)-1}{r}+\tfrac{1}{r} = \alpha (G)/r, \end{aligned}$$

as required. Here the first inequality uses the induction hypothesis twice. $\square$

We will invoke the above lemma by using the algorithm *dIS*(*G*) by Bansal et al. [3] implied by the following theorem:

#### Theorem 4

([3], Theorem 1.3) There is an $\tilde{O}(d/\log ^2 d)$ approximation algorithm *dIS*(*G*) for Independent Set on graphs of maximum degree *d* running in time $O^*(\exp (O(d)))$.

#### Proof of Theorem 1

We apply Lemma 1. By virtue of Theorem 4, *dIS*(*G*) runs in time $T(n,r)=O^*(\exp (\tilde{O}(r\log ^2 r)))$, and outputs an independent set of size at least $\alpha (G)/r$ if *G* has maximum degree *d*(*r*) for some function $d(r)=\tilde{O}(r\log ^2 r)$ with $d(r) \ge 2r$. We obtain an $O^*(\exp (\tilde{O}(n/r\log ^2 r + r\log ^2r)))$ expected time algorithm that outputs an independent set of expected size $\alpha (G)/r$.

To obtain the required probabilistic guarantee, we apply this algorithm with *r* / 3 instead of *r*. Since the size of the output is upper bounded by $\alpha (G)$ we obtain an independent set of size at least $\alpha (G)/r$ with probability at least 1 / (3*r*), and we may boost this constant positive probability using *O*(*r*) repetitions.

By Markov’s inequality these repetitions together run in $O^*(\exp (\tilde{O}(n/r\log ^2 r + r\log ^2r)))$ time with probability 3 / 4. The theorem statement follows by a union bound as these *O*(*r*) repetitions run in the claimed running time and simultaneously some repetition finds an independent set of size at least $\alpha (G)/r$, with probability at least 1 / 2. $\square$

*A deterministic algorithm* An interesting question here is whether our randomized branching algorithm can be derandomized. We show a deterministic *r*-approximation algorithm that has in slightly worse running time of $\exp (\tilde{O}(n/r \log r))$. The algorithm utilizes Feige’s algorithm [18] as a blackbox, and is deferred to Sect. 6.1.

### Graph Coloring

**Fig. 2:**
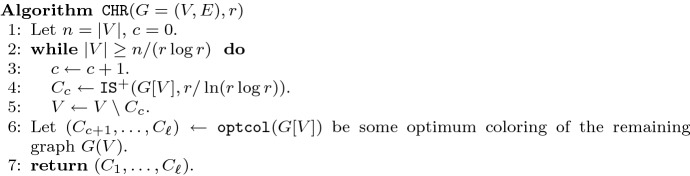
Approximation algorithm for the chromatic number

Now we use the approximation algorithm for Independent Set as a subroutine for an approximation algorithm for Coloring to prove Theorem 2 as follows (Fig. 2):

#### Proof of Theorem 2

The algorithm combines the approximation algorithm $\mathtt {IS}$ from Sect. 3.1 for Independent Set with an exact algorithm $\mathtt {optcol}$ for Coloring (see, e.g., [5]) as follows:

In the algorithm, $\mathtt {IS}^+$ denote the algorithm that makes *n* calls to $\mathtt {IS}$ and outputs the maximum size independent set found to boost the success probability. Specifically, $\mathtt {IS}^+(G[V],r/\ln (r \log r))$ clearly finds an independent set of size $\alpha (G[V])\ln (r \log r)/r$ with probability at least $1-\exp (-\varOmega (n))$, and thus it will find with at least constant positive probability in each of the at most *n* iterations an independent set of size at least $\alpha (G[V])\ln (r \log r)/r$.

We claim that $\mathtt {CHR}(G,r)$ returns with high probability a proper coloring of *G* using $\ell \le (r+2)\cdot \chi (G)$ colors. To prove the theorem, we invoke $\mathtt {CHR}(G,r-2)$ which has the same asymptotic running time. First, note that in each iteration of the while loop (Line 2 of Algorithm 2), |*V*| is decreased by a multiplicative factor of at most $1-\frac{\ln (r \log r)}{r\cdot \chi (G)}$ because *G*[*V*] must have an independent set of size at least $n/\chi (G)$ and therefore $|C_c| \ge \ln (r \log r) n/(r\cdot \chi (G))$. Before the last iteration, we have $|V| \ge n / (r \ln r)$. Thus, the number $\ell$ of iterations must satisfy

$$\begin{aligned} 1/(r \log r) \le \left( 1-\frac{\ln (r \log r)}{r\cdot \chi (G)} \right) ^{\ell -1} \le \exp \left( -\frac{\ln (r \log r)(\ell -1)}{r\cdot \chi (G)}\right) . \end{aligned}$$

This implies that $(\ell -1) \le r\cdot \chi (G)$. Consequently, the number of colors used in the first phase of the algorithm (Line 1 to Line 5) is $c \le r\chi (G)+1$. The claimed upper bound on $\ell$ follows because the number of colors used for *G*[*V*] in the second phase (Line 6) is clearly upper bounded by $\chi (G)$.

To upper bound the running time, note that Line 4 runs in time

$$\begin{aligned} \exp \left( \tilde{O}\left( \frac{n\ln (r \log r)}{r \log ^2 (r/\ln (r \log r) )}+ r\log ^2 r \right) \right) = \exp \left( \tilde{O}\left( \frac{n}{r \log r}\right) +r\lg ^2 r\right) , \end{aligned}$$

and implementing $\mathtt {optcol}(G=(V,E))$ by using the $O^*(2^{|V|})$ time algorithm from [5], Line 6 also takes $O^*(2^{n/(r \log r)})$ time and the running time follows. $\square$

Let us remark that Algorithm $\mathtt {CHR}$ uses $\exp (n/r\log r)$ space, but by using a space efficient alternative for $\mathtt {optcol}$ (see, i.e., [5]) this space usage can be reduced to $\mathrm {poly} (n)$.

### Vertex Cover and Hypergraph Vertex Cover

In this section, we show an application of the sparsification technique to Vertex Cover to obtain Theorem 3. Here the sparsification step is not applied explicitly. Instead, we utilize the sparsification Lemma of Impagliazzo et al. [25] as a blackbox. Subsequently, we solve each low-degree instance by using an algorithm of Halperin [22]. The sparsification lemma due to Impagliazzo et al. [25], shows that an instance of the *k*-Hypergraph Vertex Cover problem can be reduced to a (sub-)exponential number of low-degree instances.^7^

#### Lemma 2

(Sparsification Lemma, [9, 25]) There is an algorithm that, given a hypergraph $H=(V,{{\mathcal {E}}})$ with edges of size at most $k \ge 2$, a real number $\varepsilon > 0$, produces set systems $H_1=(V,{{\mathcal {E}}}_1),\ldots ,H_\ell =(V,{{\mathcal {E}}}_\ell )$ with edges of size at most *k* in $O^*(\ell )$ time such that

1. every subset $X \subseteq V$ is a vertex cover of *H* if and only if *X* is a vertex cover of $H_i$ for some *i*,
2. for every $i=1,\ldots ,\ell$, the degree $\varDelta (H_i)$ is at most $(k/\varepsilon )^{3k}$,
3. $\ell$ is at most $\exp (\varepsilon n)$.

The next tool is an approximation algorithm for the *k*-Hypergraph Vertex Cover problem when the input graph has low degree due to Halperin [22].

#### Theorem 5

([22]) There is a polynomial time $k-(1-o(1))\frac{k(k-1)\ln \ln \varDelta }{\ln \varDelta }$-approximation algorithm for the vertex cover problem in hypergraphs with edges of size at most *k* in which every element has degree at most $\varDelta$, for large enough $\varDelta :=\varDelta (k)$. Here *o*(1) denotes a term that tends to 0 when $\varDelta$ tends to infinity.

Now we complete the proof of the theorem by applying Lemma 2 with parameter $\varepsilon = k/(kr)^{kr}$. The number of low-degree instances $H_i$ produced by Lemma 2 is at most $\exp (\varepsilon n) = \exp \left( O\left( \frac{k}{(kr)^{kr}}\right) \right)$. Each graph $H_i$ has degree at most $\varDelta (H_i) \le (k/\varepsilon )^{3k} = (kr)^{3k^2 r}$. Note that

$$\begin{aligned} \frac{\ln \ln \varDelta (H_i)}{\ln \varDelta (H_i)} \ge \frac{\ln (3k^2 r \ln (kr))}{3k^2 r \ln (kr)} \ge \frac{1}{3k^2r}. \end{aligned}$$

Plugging this value of $\varDelta (H_i)$, Halperin’s algorithm gives the approximation factor of

$$\begin{aligned} k - (1-o(1))\frac{k(k-1)\ln \ln \varDelta }{\ln \varDelta } \le k -\tfrac{1}{2}\frac{k(k-1)}{2k^2r} \le k- \frac{1}{6r}. \end{aligned}$$

Thus this gives an $k-1/(6r)$-approximation running in time $O^*(\exp (nk/(kr)^{kr}))$ which translates to an $k-1/r$-approximation running in time $O^*(\exp (nk/(kr/6)^{kr/6}))$.

## PCP Parameters and Exponential-Time Approximation Hardness

Exponential-time approximation has connections to the trade-off questions between three parameters of PCPs: *size*, *freebit*, and *gap*. To formally quantify this connection, we define new terms, formally illustrating the ideas that have been already around in the literature. We define a class of languages FGPCP which stands for *Freebit and Gap-amplifiable PCP*. Let *c* and *g* be positive reals, and *S*, *F* be non-decreasing functions. A language *L* is in $\text{ FGPCP } _{c}(S, F)$ if there is a constant $g_0 >1$ such that, for all constants $g \ge g_0$, there is a verifier $V_{g}$ that, on input $x \in \{0,1\}^n$, has access to a proof $\pi : |\pi | = O(S(n,g))$ and satisfies the properties:

- The verifier runs in $2^{o(n)}$ time.
- If $x \in L$, then there is a proof $\pi$ such that $V^{\pi }_g(x)$ accepts with probability $\ge c$.
- If $x \not \in L$, then for any proof $\pi$, $V^{\pi }_g(x)$ accepts with probability $\le c/g$.
- For each *x* and each random string *r*, the verifier has $\le F(g)$ accepting configurations.

The parameters *g*, *S* and $\log F$ are referred to as *gap*, *size* and *freebit* of the PCPs respectively. For convenience, we call *F*(*g*) the *freeness* of the PCP. Intuitively, ones may view FGPCP as a class of PCPs parameterized by gap *g*. An interesting question in the PCPs and hardness of approximation literature has been to find the smallest functions *S* and *F*. Roughly speaking, if one can construct a small PCP with small freeness, this could be turned into a stronger lower bound on the running time. The following theorem made this intuition precise.

### Theorem 6

Let $r >1$ be a constant. If $\mathsf{SAT} \in \text{ FGPCP } _\delta (S, F)$ for some function *S* such that $\frac{S(n,r)}{n}$ is a non-decreasing function in *n*,^8^ then an *r*-approximation algorithm for Independent Set, on graph *G*, requires the running time of $2^{\varOmega (S^{-1}(|V(G)|,r)/r F(r))}$ unless ETH fails.

Here the connection between PCP size and running time lower bound is captured by the term $S^{-1}$. When *r* is fixed and *S* is an increasing function, then $S^{-1}(\cdot , r)$ is well-defined (e.g., the size function such as $S(n,r) = n \log r$ has an inverse $S^{-1}(N,r) = N/ \log r$).

We prove the theorem later in this section. Meanwhile, we argue that the trade-off result follows directly.

### Corollary 2

Assuming that SAT has no $2^{o(n)}$-time randomized algorithm and that $\mathsf{SAT} \in \text{ FGPCP } _{\delta }(S,F)$, then it must be the case that $S(n,g) \cdot F(g) = \varOmega (n \cdot \frac{\log ^2 g}{\mathsf{poly}( \log \log g)})$.

### Proof

Assume otherwise that such a PCP exists with the parameters *S* and *F* such that $S(n,g) F(g) = o(n \cdot \frac{\log ^2 g}{\mathtt {poly} (\log \log g)})$. Denote |*V*(*G*)| by *N*. Notice that $S^{-1}(N,r) =\omega (N \cdot \frac{F(r) \mathsf{poly}( \log \log r)}{\log ^2 r})$, and Theorem 6 would imply that there is no $2^{\omega (|V(G)| \cdot \frac{\mathsf{poly}( \log \log r)}{r \log ^2 r})}$, contradicting the existence of our approximation algorithm for the maximum independent set problem. $\square$

Now let us phrase the known PCPs in our framework of FGPCP. Chan’s PCPs [12] can be stated that $\mathsf{SAT} \in \text{ FGPCP } _{1-o(1)}(\mathrm {poly}, O(\log g))$. Applying our results, this means that if one wants to keep the same freebit parameters given by Chan’s PCPs, then the size must be at least $\varOmega (n \log g)$. Another interesting consequence is a connection between Vertex Cover and Freebit PCPs in the polynomial time setting [4].

### Theorem 7

([4]—Section 7 on the “reverse connection”) Vertex Cover is $(2-\epsilon )$ hard to approximate if and only if $\mathsf{SAT} \in \mathsf{FGPCP}_{1/2 -\epsilon }(\mathsf{poly}, 1)$.

The intended PCPs in Theorem 7 have arbitrary small soundness while the freeness remains 1. Our Corollary 2 implies that such a PCP must have size at least $\varOmega (n \log ^2 g)$.

### Proof of Theorem 6

First, we define a standard terminology for dealing with *constraint satisfaction problems (*CSP*)*. An input to the general CSP is a collection of clauses $C_1,\ldots , C_m$ over *n* variables $x_1,\ldots , x_n$ where each clause is a predicate over some subset of variables. Given a CSP $\phi$, the *value* of $\phi$, denoted by $\mathsf{val}(\phi )$ is the maximum number of clauses that can be simultaneously satisfied by an assignment. The goal of the problem is to compute the value of an input CSP. For each clause $C_i$, let $Y_i \subseteq \{x_1,\ldots , x_n\}$ be the set of variables appearing in $C_i$. The *freeness* of $\phi$ is the maximum, over all clauses $C_i$, of the number of ways $C_i$ can be satisfied by different assignments on variables in $Y_i$.

*Step 1: Creating a hard CSP* We will need the following lemma that creates a “hard” CSP from FGPCP. This CSP will be used later to construct a hard instance of Independent Set.

#### Lemma 3

If $\mathsf{SAT} \in \text{ FGPCP } _{\delta }(S,F)$, then, for any $g>1$, there is a randomized reduction from an *n*-variable SAT$\phi$ to a CSP$\phi '$ having the following properties (w.h.p.):

- The number of variables of $\phi '$ is $\le S(n)$.
- The number of clauses of $\phi '$ is $\le 10 S(n) g/\delta$.
- The freeness of $\phi '$ is $\le F(g)$.
- If $\phi$ is satisfiable, then $\mathsf{val}(\phi ') \ge \delta /2$. Otherwise, $\mathsf{val}(\phi ') \le 6\delta /g$.

#### Proof

Let *g* be any number and $V_g$ be the corresponding verifier. On input $\phi$, we create a CSP$\phi '$ as follows. For each proof bit $\Pi _i$, we have variable $x_i$. The set of variables is $X = \{x_1,\ldots , x_{S(n)}\}$. We perform $M = 10 \lceil S(n)g/\delta \rceil$ iterations. In iteration *j*, the verifier picks a random string $r_j \in \{0,1\}^R$ where *R* is the random coins used by the verifier and create a predicate $P_j(x_{b_1},\ldots , x_{b_q})$, where $b_1,\ldots , b_q$ are the proof bits read by the verifier $V_g^{\Pi }$ on random string $r_j$. This predicate is true on assignment $\gamma$ if and only if the verifier accepts the local assignment where $\Pi _{b_i} = \gamma (x_i)$ for all $i \in [q]$.

First, assume that $\phi$ is satisfiable. Then there is a proof $\Pi ^*$ such that the verifier $V^{\Pi ^*}(\phi )$ accepts with probability $\delta$. Let $\gamma : X \rightarrow \{0,1\}$ be an assignment that agrees with the proof $\Pi ^*$. So, $\gamma$ satisfies each predicate $P_j$ with probability $\delta$, and therefore, the expected number of satisfied predicates is $\delta M$. By Chernoff’s bound, the probability that $\gamma$ satisfies less than $\frac{\delta M}{2}$ predicates is at most $2^{-\delta M/ 8} \le 2^{-n}$.

Next, assume that $\phi$ is not satisfiable. For each assignment $\gamma : X \rightarrow \{0,1\}$, the fraction of random strings satisfied by the corresponding proof $\Pi _{\gamma }$ is at most $\delta /g$. When we pick a random string $r_j$, the probability that $V^{\Pi _{\gamma }}(\phi , r_j)$ accepts is then at most $\delta /g$. So, over all the choices of *M* strings, the expected number of satisfied predicates is $\delta M/g \ge 10 S(n)$. By Chernoff’s bound, the probability that $\gamma$ satisfies more than $\delta M/g$ predicates is at most $2^{-10 S(n)}$. By union bound over all possible proofs of length *S*(*n*) (there are $2^{S(n)}$ such proofs), the probability that there is such a $\gamma$ is at most $2^{S(n)} 2^{-10 S(n)} \le 2^{- S(n)}$. $\square$

*Step 2: FGLSS reduction* The FGLSS reduction is a standard reduction from CSP to Independent Set introduced by Feige et al. [19]. The reduction simply lists all possible configurations (partial assignment) for each clause as vertices and adding edges if there is a conflict between two configuration. In more detail, for each predicate $P_i$ and each partial assignment $\gamma$ such that $P_i(\gamma )$ is true, we have a vertex $v(i,\gamma )$. For each pair of vertices $v(i,\gamma ) v(i', \gamma ')$ such that there is a variable appearing in both $P_i$ and $P_{i'}$ for which $\gamma (x_j) \ne \gamma '(x_{j})$, we have an edge between $v(i,\gamma )$ and $v(i', \gamma ')$.

#### Lemma 4

(FGLSS Reduction [19]) There is an algorithm that, given an input CSP$\phi$ with *m* clauses, *n* variables, and freeness *F*, produces a graph $G=(V,E)$ such that (i) $|V(G)| \le m F$ and (ii) $\alpha (G) = \mathsf{val}(\phi ) m$, where $\mathsf{val}(\phi )$ denotes the maximum number of predicates of $\phi$ that can be satisfied by an assignment.

*Combining everything* Assume that $\mathsf{SAT} \in \text{ FGPCP } _{\delta }(S, F)$. Let $g >0$ be a constant and $V_g$ be the verifier of SAT that gives the gap of *g*. By invoking Lemma 3, we have a CSP$\phi _1$ with *S*(*n*, *g*) variables and $100 S(n,g) g/\delta$ clauses. Moreover, the freeness and gap of $\phi _1$ are *F*(*g*) and *g*, respectively. Applying the FGLSS reduction, we have a graph *G* with $N=|V(G)| = 100 S(n,g) F(g) g/\delta = O(S(n,g) F(g) g)$. Now assume that we have an algorithm ${{\mathcal {A}}}$ that gives a *g*-approximation algorithm in time $2^{\frac{o(S^{-1}(N,g))}{g F(g)}}$. Notice that $S^{-1}(N,g) \le O(n g F(g))$ (here we used the assumption that *S* is at least a linearly growing function.) and therefore algorithm ${{\mathcal {A}}}$ distinguishes between Yes- and No-instance in time $2^{o(n)}$, a contradiction.

*Hardness under Gap-ETH* Dinur [17] and Manurangsi and Raghavendra [32] made a conjecture that SAT does not admit an approximation scheme that runs in $2^{o(n)}$ time. We observe a Gap-ETH hardness of *r*-approximating Independent Set in time $2^{n/r^c}$ for some constant *c*. The proof uses a standard amplification technique and is deferred to Sect. 6.2.

## Further Research

Our work leaves ample opportunity for exciting research. An obvious open question is to derandomize our branching, e.g., whether Theorem 1 can be proved without randomized algorithms. While the probabilistic approximation guarantee can be easily derandomized using splitters, it seems harder to strengthen the expected running time bound to a worst-case running time bound.

Can we improve the running times of the other algorithms mentioned in the introduction that use the partition argument, possibly using the randomized branching strategy? Specifically, can we $(1+\varepsilon )$-approximate Independent Set on planar graphs in time $O^*(2^{(1/\varepsilon )/\log (1/\varepsilon )})$, or *r*-approximate Independent Set in time $O^*(2^{tw/r \log r})$? As mentioned in the introduction, a result of Marx [34] still leaves room for such lower order improvements. Another open question in this category is how fast we can *r*-approximate *k*-Independent Set, where the goal is to find an independent set of size of *k*. Recently, Chalermsook et al. [10] showed, under the Gap-ETH assumption, that finding a *k*-Independent Set always takes time $n^{\varOmega (k)}$, despite assuming the existence of *q*-clique for $q>> k$. It remains open whether one can rule out such an algorithm under ETH. Finally, a big open question in the area is to find or exclude a $(2-\varepsilon )$-approximation for Vertex Cover in graphs in subexponential time for some fixed constant $\varepsilon >0$. We remark that the dependence on $\epsilon$ presented in our paper has recently been improved by Manurangsi and Trevisan [33].

## Omitted Results

### A Deterministic Algorithm for Independent Set

In this section, we give a deterministic *r*-approximation algorithm that runs in time $2^{\tilde{O}(n/ r \log r)}$. This algorithm is a simple consequence of Feige’s algorithm [18], that we restate below in a slightly different form.

#### Theorem 8

([18]) Let *G* be a graph with independence ratio $\frac{\alpha (G)}{|V(G)|} = 1/k$. Then, for any parameter $t \in {{\mathbb {N}}}$, one can find an independent set of size $t \cdot \lfloor \log _k (\frac{n}{6kt}) \rfloor$ in time $k^{O(t)} \cdot \mathrm {poly} (n)$.

Feige used the above theorem with parameter $k= \mathsf{poly} \log n$, and $t = \Theta (\log n / \log \log n)$, so he obtained an algorithm that runs in polynomial time. Here we will be using the power of his algorithm in (mildly) exponential time.

- If $\alpha (G) < \frac{n}{\log ^2 r}$, then we can enumerate all independent sets of size $n/(r \log ^2 r)$ (this is an *r*-approximation) in time ${n \atopwithdelims ()n/(r \log ^2 r)} \le (e r \log ^2 r)^{\frac{n}{r \log ^2 r}} \le 2^{O(n/(r \log r))}$.
- Otherwise, the independence ratio is at least 1 / *k* where $k = \log ^2 r$. We choose $t = \lceil n/(r \log r) \rceil$, so Feige’s algorithm finds an independent set of size at least
  $$\begin{aligned} t \cdot \log _k \left( \frac{n}{6kt}\right) = \varOmega \left( \frac{n}{ r\log r} \cdot \log _k (r \log r)\right) = \varOmega (n/(r \log \log r)) \end{aligned}$$
  The running time of this algorithm is
  $$\begin{aligned} k^{O(t)} \mathrm {poly} (n) = 2^{O\left( \frac{n(\log \log r)}{r \log r}\right) } \end{aligned}$$
  If we redefine $r'= r \log \log r$, then the algorithm is an $r'$-approximation algorithm that runs in time $2^{O(n (\log \log r')^2/r' \log r')}$.

### Gap-ETH Hardness of Independent Set (Sketch)

We now sketch the proof. We are given an *n*-variable 3-CNF-SAT formula $\phi$ with perfect completeness and soundness $1-\epsilon$ for some $\epsilon >0$. We first perform standard amplification and sparsification to get $\phi '$ with gap parameter *g*, the number of clauses is *ng*, and freeness is $g^{O(1/\epsilon )}$. Then we perform FGLSS reduction to get a graph *G* such that $|V(G)| = n g^{O(1/\epsilon )}$. Therefore, *g*-approximation in time $2^{o(|V(G)|/g^{O(1/\epsilon )})}$ would lead to an algorithm that satisfies more than $(1-\epsilon )$ fraction of clauses in 3-CNF-SAT formula in time $2^{o(n)}$. In other words, any $2^{n/r^c}$-time algorithm that *r*-approximates Independent Set can be turned into a $(1+O(1/c))$-approximation algorithm for approximating 3-CNF-SAT in sub-exponential time.

## Footnotes

1 That is, given a graph and integer *k* answer YES if it has an independent set of size at least 2*k* and NO if it has no independent set of size at least *k*.

2 This observation was already made by Bourgeois et al. [8], but we exploit it in a new way.

3 That is, restricting ourselves to the set of variables $\{x_i\}_{i \in B}$ queried by the verifier, freeness is the number of possible assignments to these variables that cause the verifier to accept.

4 Roughly speaking, the existence of a PCP with freeness *F*(*g*) (where *g* is a gap) implies $\varOmega (\frac{d}{F(d) \log ^3 d})$ hardness of approximating independent set in degree-*d* graphs.

5 In fact, Moshkovitz and Raz give a construction of nearly linear-size 2-query PCPs with sub-constant error; this can be composed with the results of, e.g., [23, 24, 37] to get a PCP with low freebit complexity. The above parameters are taken from the composition with [37].

6 The problem is also known under other names, such as *k*-Hitting Set and Hypergraph Transversal in *k*-uniform hypergraphs

7 The original formulation is for the Set Cover problem and the most popular formulation is for CNF-SAT problem, but they are all equivalent by direct transformation.

8 Since we think of *r* as a fixed number, the function *S*(*n*, *r*) should be seen as a function on a single variable *n*. We will focus on the size functions *S* that are invertible over the domain of sufficiently large natural numbers. This is generally true for polynomial functions and near-linear functions (such as $S(n,r) = n \log ^2 n \log r$).
